# Electrical Impedance Tomography-Based Abdominal Subcutaneous Fat Estimation Method Using Deep Learning

**DOI:** 10.1155/2020/9657372

**Published:** 2020-06-11

**Authors:** Kyounghun Lee, Minha Yoo, Ariungerel Jargal, Hyeuknam Kwon

**Affiliations:** ^1^Center for Mathematical Analysis and Computation, Yonsei University, Seoul 03722, Republic of Korea; ^2^National Institute for Mathematical Science, Daejeon 34047, Republic of Korea; ^3^Department of Computational Science and Engineering, Yonsei University, Seoul 03722, Republic of Korea; ^4^College of Science and Technology, Yonsei University, Wonju 26493, Republic of Korea

## Abstract

This paper proposes a deep learning method based on electrical impedance tomography (EIT) to estimate the thickness of abdominal subcutaneous fat. EIT for evaluating the thickness of abdominal subcutaneous fat is an absolute imaging problem that aims at reconstructing conductivity distributions from current-to-voltage data. Existing reconstruction methods based on EIT have difficulty handling the inherent drawbacks of strong nonlinearity and severe ill-posedness of EIT; hence, absolute imaging may not be possible using linearized methods. To handle nonlinearity and ill-posedness, we propose a deep learning method that finds useful solutions within a restricted admissible set by accounting for prior information regarding abdominal anatomy. We determined that a specially designed training dataset used during the deep learning process significantly reduces ill-posedness in the absolute EIT problem. In the preprocessing stage, we normalize current-voltage data to alleviate the effects of electrodeposition and body geometry by exploiting knowledge regarding electrode positions and body geometry. The performance of the proposed method is demonstrated through numerical simulations and phantom experiments using a 10 channel EIT system and a human-like domain.

## 1. Introduction

Abdominal obesity is closely linked to metabolic syndrome and cardiovascular diseases [1–3]. As a major health indicator, it is desirable to estimate the regional distribution of abdominal fats, such as subcutaneous and visceral fats. Computed tomography (CT) and magnetic resonance imaging can quantitatively estimate the distribution of abdominal fat [4]. However, these methods are expensive and unsuitable for daily use. Furthermore, CT has associated safety issues based on radiation exposure [5]. Therefore, there is a growing demand for a cheaper and safer abdominal fat evaluation method that is practical for continuous self-monitoring to track body fat status as part of a daily routine.

Electrical impedance tomography (EIT) [6–8] may be the top candidate for meeting this demand based on its low economic burden, continuous self-monitoring capabilities, and suitability for daily routines. EIT aims at visualizing the distributions of electrical conductivity inside the human body. Such distributions can be used to evaluate the thickness of subcutaneous fat because the electrical properties of adipose tissue are significantly different from those of other tissues [9–11]. EIT uses a number of electrodes (typically 8 to 32) attached to the surface of the body. Current-voltage data are acquired by applying alternating currents over various frequencies (from tens of kilohertz to megahertz) and measuring voltages through the attached electrodes. These voltages reflect internal conductivity distributions. Here, the amount of current injected to human body is less than or equal to 10 mA at the current excitation frequency 100 kHz, and the safe range of current depends on the excitation frequency [12]. Conductivity distributions are recovered from the current-voltage data using reconstruction algorithms. It is theoretically guaranteed that a conductivity distribution can be uniquely identified based on (infinite) current-voltage data [13–15]. The EIT imaging reconstruction problem of recovering conductivity distributions inside the abdomen, which is referred to as the absolute imaging problem [16], is highly nonlinear and severely ill-posed. Despite over 30 years of development of EIT reconstruction methods, performance for absolute imaging is still insufficient for clinical applications, although difference imaging has been successful in some applications as a contrast imaging method [6, 17]. There have been numerous studies on EIT reconstruction algorithms, such as backprojection [18], NOSER [19], GREIT [20], the D-bar method [21], factorization method [22], and regularized least squares method [23]. Representative methods for solving the absolute imaging problem are the regularized least squares method [6, 19, 24] and D-bar method [25–27]. The regularized least squares method is based on the minimization problem argmin *γ*1/2‖*F*(*γ*) − V‖^2^ + Reg(*γ*) for recovering a conductivity distribution *γ* from given data **V**, where *F* is a forward operator that maps data from *γ* to data in **V**(i.e., *F* : *γ*⟶**V**), where Reg(*γ*) is a regularization term and *k* · *k* is the Euclidean norm. These methods handle ill-posedness by forcing the minimizer to have a desired property that is determined based on prior knowledge regarding *γ* and incorporated in Reg(*γ*). However, this approach fails to produce useful images for absolute abdominal imaging with regularization, including *L*_2_ and *L*_1_ regularization, and variation [6] (see Section 2.3). The D-bar method performs nonlinear direct reconstruction, but reconstruction can be inaccurate when data are measured for only a small portion of the boundary [28].

When describing the forward operator *F*, most conventional methods use a physics-based model. Specifically, a generalized Laplace equation representing electric potential distributions at low frequencies is typically developed from Maxwell's equation [6, 7]. Physics-based models not only depend on the relationships between conductivity distributions and current-voltage data, but also on geometrical factors, body shape, and electrode positions [29]. Therefore, errors or uncertainty in geometry factors can easily cause the reconstruction process to misinterpret the underlying current-voltage data, thereby compromising image reconstruction results. In fact, the influence of geometrical factors is so severe that regularization in physics-based models is not sufficient for absolute imaging [27, 30–32]. To handle this undesirable influence, it would be advantageous to incorporate geometrical factors in the regularization term Reg(·). However, this is a difficult proposition for physics-based models based on the difficulty of explicitly describing geometrical influences on data during regularization.

To avoid using physics-based models, which are the fundamental cause of many of the difficulties in absolute imaging, we propose using a deep learning technique that incorporates a data-based model. Recently, deep learning methods have been actively applied to EIT [33]. Such methods can be divided into two main types: methods that map images to images and methods that map data to images. The former type enhances the resolution of relatively low-resolution images generated by conventional methods [27, 34]. The latter type directly learns mappings from measured data to images [35, 36]. In this study, we developed a method of the second type by adopting a multilayer perceptron (MLP), which is one of the deep-learning techniques. MLPs use fully connected layers, meaning each node in each interior layer is connected to all nodes in the next layer [37]. This fully connected structure is suitable for EIT because boundary voltage data are entangled with the global structures of conductivity distributions [7].

Furthermore, we combine an MLP with a special data normalization technique to reduce inherent geometrical influences. We establish an operator *G* : **V**⟶*γ* that can be considered as a backward operator corresponding to the forward operator *F* by using a training dataset consisting of geometrical dependency-reduced voltage data (Section 3.2). Specifically, we normalize the measured voltage **V** to minimize geometrical dependency and apply this normalized voltage to the MLP using a normalization map (Section 3.1). When generating a training dataset D, we use simplified conductivity distributions by layering imaging domains of uniform thickness (Section 3.3). This simplification makes the estimation of the thickness of subcutaneous fat much easier and reduces the ill-posedness of the absolute imaging problem by reducing the number of unknown variables.

This remainder of this paper is organized as follows. In Section 2, we present preliminary information, including the electrical properties of the abdomen, data acquisition methods, and conventional methods. The proposed method is introduced in Section 3, which has three subsections focusing on data normalization, the MLP, and training datasets. In Section 4, we present numerical simulation results. Finally, we conclude this paper in Section 5.

## 2. Preliminary Study: Conductivity and Data

### 2.1. Electrical Properties of the Abdomen

**Figure 1:**
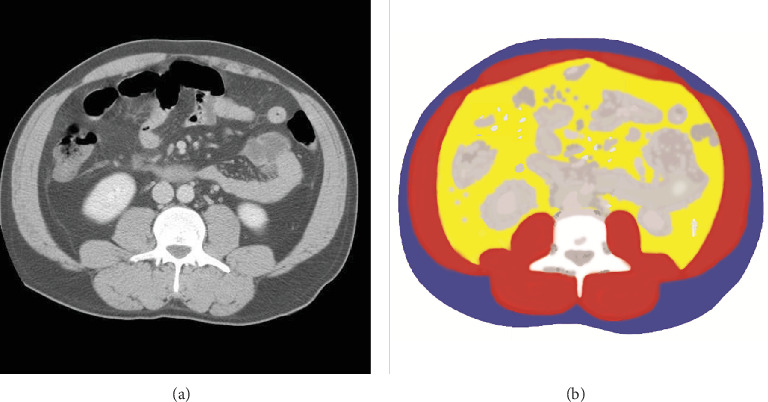
Abdominal CT image (a) and corresponding segmented image (b) separated into three main regions (subcutaneous fat colored blue, muscle colored red, and visceral fat colored yellow) with bone (white) and other tissues (gray).

**Figure 2:**
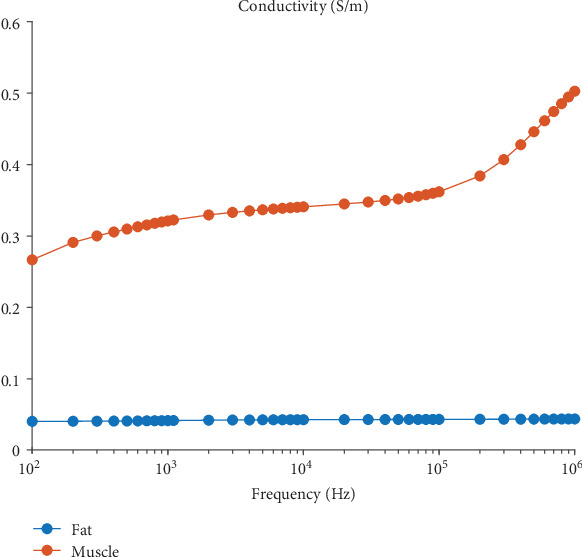
Conductivity values of subcutaneous fat (blue) and muscle (red) tissues over the frequency range of 100 Hz to 1 MHz [38].

The abdomen has different electrical properties related to different organs and can be roughly divided into four regions: subcutaneous fat (just below the skin), abdominal muscle, visceral fat (fat that surrounds the organs), and organ tissue, as shown in Figure 1. Fat and muscle tissues have very distinct conductivity spectra over the frequencies plotted in Figure 2 [38], which reveal that the conductivity of muscle tissue is six times greater than that of fat. Let *γ*_*f*_ and *γ*_*m*_ denote the conductivity values of fat and muscle, respectively. Then, we assume that

(1) $$\begin{array}{c} \gamma_{f}<\gamma_{m}\leq 10\gamma_{f}. \end{array}$$

These distinct electrical properties of organs in the abdomen motivate the use of impedance data and EIT techniques for the estimation of subcutaneous fat thickness by distinguishing fat from muscle.

### 2.2. Data Acquisition

Let an imaging object occupy a two- or three-dimensional space *Ω* bounded by its surface *∂Ω*. The conductivity in *Ω* at an angular frequency *ω* and position **r** = (*x*, *y*) or (*x*, *y*, *z*) is denoted by *γ*(**r**). A set of surface electrodes attached to *∂Ω* apply currents and measure corresponding voltages. When applying a sinusoidal current between a chosen pair of electrodes at an angular frequency *ω*, the induced voltage in the body can be expressed as *U*(**r**)sin(*ωt* + *θ*(*ω*, **r**)), where *θ* indicates the phase angle. Then, the corresponding time-harmonic potential $u\left(r\right)=U\left(r\right)\exp\left(\theta \left(\omega ,r\right)\sqrt{-1}\right)$ is governed by

(2) $$\begin{array}{c} \left\{\begin{array}{c} \nabla \cdot \left(\gamma \nabla u^{\gamma }\right)=0\text{ in }\Omega ,\\ n\cdot \left(\gamma \;u\nabla^{\gamma }\right)=g\text{ on }\partial \Omega , \end{array}\right. \end{array}$$

where **n** is the outward unit normal vector corresponding to *∂Ω* and *g* is the boundary current density on *∂Ω* induced by the applied current.

Let (*ε*_1_, *ε*_2_, ⋯, *ε*_*E*_) denote the attached surface electrodes. We sequentially apply *M* different currents using chosen electrode pairs {(*ε*_1_+__, *ε*_1_−__), (*ε*_2_+__, *ε*_2_−__), ⋯, (*ε*_M_+__, *ε*_M_−__)}, where 1_±_, ⋯, *M*_±_ ∈ {1, 2, ⋯, *E*}. Let *u*_*j*_ denote the induced potential in (2) corresponding to the *j*th applied current, where a sinusoidal current of *I* mA at an angular frequency *ω* is applied through the electrode pair (*ε*_*j*_+__, *ε*_*j*_−__). By denoting the corresponding Neumann data as *gj*, we have ∫_*ε*_*j*_+___*g*_*j*_*ds* = *I* = −∫_*ε*_*j*__*g*_*j*_*ds*, where *gj* is approximately zero on *∂Ω*(E_*j*_+__ ∪ E_*j*_−__), where *ds* is the surface element. To estimate a conductivity distribution, we measure the voltage difference between the *i*th pair of electrodes subject to the *j*th applied current as

(3) $$\begin{array}{c} V_{j,i}=u_{j}^{\gamma }\left|\varepsilon_{i_{+}}-u_{j}^{\gamma }\right|\varepsilon_{i_{-}}, \end{array}$$

for *j*, *i* = 1, 2 ⋯ , *M*. We do not use *V*_*j*,*i*_ for (*j*, *i*) as εj+,εj−∩εi+,εi−≠0 based on the uncertainty caused by skin-electrode contact impedances [7, 39, 40].

**Figure 3:**
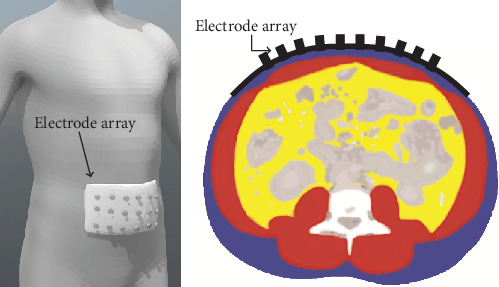
Left image shows an illustrative human with an electrode array attached to their abdomen in 3D. The right image shows a cross section of the left image with the electrode array depicted in black. In the right image, blue, red, yellow, white, and gray colors represent subcutaneous fat, muscle, visceral fat, bone, and other tissues, respectively.

The voltage *V*_*j*,*i*_ reflects the conductivity distribution *γ* according to the following relation:

(4) $$\begin{array}{c} V_{j,i}\approx \frac{1}{I}\int_{\Omega }\gamma \left(\text{r}\right)\nabla u_{j}^{\gamma }\left(\text{r}\right)\cdot \nabla u_{i}^{\gamma }\left(\text{r}\right)d\text{r}, \end{array}$$

where *d ***r** is the area element. The voltage *V*_*j*,*i*_ heavily depends on the body geometry *Ω* and electrode positions (*ε*_1_, *ε*_2_, ⋯, *ε*_E_), which are difficult to acquire in practice. To minimize the dependency on body geometry and electrode positions, we fix the positions of the electrodes on a curved plate, as shown in Figure 3. The shape of the curved plate is predetermined and designed to fit the human abdomen. By concatenating all voltages *V*_*j*,*i*_ in order, we generate a vector **V** of *V*_*j*,*i*_ values as follows:

(5) $$\begin{array}{c} V={\left[V_{j1}{,}_{i1},V_{j2}{,}_{i2},\cdots ,V_{j_{M*},i_{M_{*}}}\right]}^{T}, \end{array}$$

where (*j*_1_, *i*_1_), (*j*_2_, *i*_2_), ⋯, (*j*_*M*∗_, *i*_*M*_∗__) are the ordered index pairs of j,i: εj+,εj−∩εi+,εi−=0 and *M*_∗_ is the number of voltages in **V**. Then, **V** is referred to as a vector of current-voltage data.

### 2.3. Conventional Method: Sensitivity Approach

The most widely used EIT image reconstruction method is the sensitivity method [19], which operates based on the voltage-conductivity relationship in (4). This method requires a discretized imaging domain *Ω* with *L* subregions (mostly triangular) *Ω*_1_, *Ω*_2_, ⋯, *Ω*_*L*_, which can be defined as

(6) $$\begin{array}{c} \Omega =\cup_{\ell =1}^{L}\Omega_{\ell }. \end{array}$$

Assuming that *γ* is constant in each subregion *Ω*, the voltage in (4) can be expressed approximately as a linear system **V** = *𝕊*_*γ*_*γ* for *γ* = [*γ* | *Ω*_1_, *γ* | *Ω*_2_,⋯,*γ* | *Ω*_*L*_]^*T*^ Where *𝕊γ* is an *M*_∗_ × *L* sensitivity matrix defined as (*𝕊*_*γ*_)_*α*,*β*_ = 1/*I*∫_*Ω*_*ℓ*__∇*u*_*j*_^*γ*^(**r**) · ∇*u*_*j*_^*γ*^(**r**)*d ***r** with *α* = (*j*, *i*), *β* = *ℓ*. *γ* can be derived from **V** by solving the linear system **V** = *𝕊*_*γ*_*γ*. However, the matrix *𝕊γ* is ill-conditioned. Therefore, to recover *γ* from **V**, the regularized least squares method [6] is used as follows:

(7) $$\begin{array}{c} \text{V}\mapsto \gamma ≔\underset{\gamma }{\text{argmin}}\frac{1}{2}{\left\|S_{\gamma }\gamma ‐\text{V}\right\|}^{2}+\lambda_{R}\text{Reg}\left(\gamma \right), \end{array}$$

where ‖·‖ is the standard Euclidean norm, Reg is a regularization operator, and *λ*_*R*_ > 0  is a regularization parameter. Tikhonov and total variation regularizations are also widely used [19, 40]. However, the regularized least squares method for absolute EIT suffers from the fundamental difficulty in handling the ill-conditioned matrix S*γ* based on the unknown *γ* and forward modelling errors.

## 3. Method: Absolute EIT Reconstruction Using Deep Learning

In this section, we discuss the proposed absolute image reconstruction algorithm for estimating subcutaneous fat thickness. We develop a map *G* from **V** to *γ* based on deep-learning techniques combined with the special data normalization method introduced in [11]. The map *G* is defined by two other maps, namely, the data normalization map Ψ and conductivity reconstruction map *Ξ*, as follows:

(8) $$\begin{array}{c} G=\Xi \circ \Psi . \end{array}$$

In Section 3.1, we presented a data normalization map Ψ from the data **V** to $\hat{V}$, which minimizes forward modelling error as follows:

(9) $$\begin{array}{c} \Psi :\text{V}\mapsto \hat{V}. \end{array}$$

In Sections 3.2 and 3.3, for reconstructing the conductivity *γ* from the normalized data $\hat{V}$, we introduced a map *Ξ*, which is defined as

(10) $$\begin{array}{c} \Xi :\hat{V}\mapsto \gamma , \end{array}$$

based on deep learning techniques.

### 3.1. Normalization of Current-Voltage Data

The reconstruction of the conductivity distribution *γ* from the data **V** is very sensitive to forward modelling errors caused by the inaccuracy of electrode positions and geometrical uncertainty [7]. To alleviate such forward modelling errors, we normalize the data **V** based on information regarding boundary geometry. The map Ψ is designed to minimize the geometry dependency of the data *V*_*j*,*i*_, which are components of **V**, as follows ([11], the equation (2.18)):

(11) $$\begin{array}{c} \Psi \left(\text{V}\right)=\hat{V},\text{where }{\hat{V}}_{j,i}≔\frac{S_{j,i}}{{\text{V}}_{j,i}}\approx \frac{\int_{\Omega }\nabla v_{j}\left(\text{r}\right)\cdot \nabla v_{i}\left(\text{r}\right)d\text{r}}{\int_{\Omega }\gamma \left(\text{r}\right)\nabla u_{j}^{\gamma }\left(\text{r}\right)\cdot \nabla u_{i}^{\gamma }\left(\text{r}\right)d\text{r}}, \end{array}$$

and *S*_*j*,*i*_ = 1/*I*∫_*Ω*_∇*v*_*j*_(**r**) · ∇*v*_*i*_(**r**)*d ***r**, where *v*_*j*_ is the solution to *v*_*j*_ = 0 in *Ω* with the boundary condition **n** · ∇*v*_*j*_ = *g*_*j*_ on *∂Ω*. Here, ${\hat{V}}_{j,i}$ can be considered as a weighted harmonic average of conductivity whose weight depends on the distribution of ∇*u*_*j*_^*γ*^ · ∇*u*_*i*_^*γ*^. It should be noted that if the conductivity distribution *γ* is homogeneous and represented as a constant, then $u_{j}^{\gamma }\left(\text{r}\right)=v_{j}\left(\text{r}\right)/\gamma \left(\text{r}\right)\text{ and }{\hat{V}}_{j,i}=\gamma$ for all *i*, *j*, regardless of the electrode positions and boundary geometry.

### 3.2. Multilayer Perceptron

We reconstruct the conductivity distribution *γ* from the normalized data $\hat{V}$ using an MLP, which is a deep-learning technique that can capture nonlinear relationships between input and output data [41, 42]. An MLP can serve as a tool for creating a representation function based on a given set of credible pairs of input and output data, which is referred to as a training dataset. This representation functionality can be considered as an inverse solver for EIT. We recover the conductivity *γ* from the normalized voltage data **V** by constructing a mapping $\Xi :\hat{V}⟶\gamma$ using an MLP. The map *Ξ* is constructed by compositing multiple linear and nonlinear functions called activation functions. Below, we provide details regarding how *Ξ* is constructed from linear and nonlinear functions.

**Figure 4:**
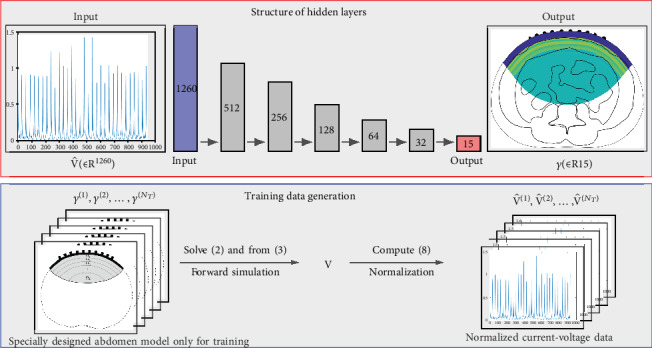
Schematic diagram of the MLP used in this study. To apply the MLP, we require a training dataset, as shown in the blue box. In the red box, we illustrate the details of the MLP structure. The numbers in the black box indicate numbers of nodes. The inputs are obtained from the training dataset and the outputs are conductivity values.

An MLP consists of several layers of nodes or neurons, as shown in Figure 4. To apply an MLP to EIT, we define the nodes in the input layer as the normalized voltages in the vector **V** and the nodes in the output layer as the conductivity values *γ* for each subregion *Ω* bin (6). The hidden layers, which are neither input nor output layers, are used to extract complex features from the relationships between the conductivity and voltage data. Let an MLP contain *J* layers and let the *j*th layer contain *N*_*j*_ nodes. Since the nodes in the first layer (input layer) are measured voltage data and the nodes in the last layer (output layer) are the conductivity values from subregions, we have *N*_1_ = *M*_∗_ and *N*_*J*_ = *L*. The representation function *Ξ* in the MLP has the following form of successive compositions:

(12) $$\begin{array}{c} \Xi \left(\hat{V}\right)=h_{J-1}\circ \cdots \circ h_{2}\circ h_{1}\left(\hat{V}\right), \end{array}$$

where *h*_*j*_ : ℝ^*N*_*j*_^⟶ℝ^*N*_*j*+1_^ is given by

(13) $$\begin{array}{c} h_{j}\left({\text{o}}^{j}\right)={\left[o_{1}^{j+1},o_{2}^{j+1},\cdots ,o_{N_{J+1}}^{j+1}\right]}^{T}\text{ with }o_{m}^{j+1}=\xi \left(\overset{N_{j}}{\underset{n=1}{\sum }}W_{m,n}^{j}\;o_{n}^{j}\right) \end{array}$$

for **o**^*j*^ ∈ ℝ^*N*_*j*_^. Here, *W*_*m*,*n*_^*j*^ is the weight connecting the *m*th node (neuron) in the (*j* + 1)th layer to the *n*th node (neuron) in the *j*th layer and *ξ* is an activation function. It should be noted that **o**^1^ are the input data, meaning $o^{1}=\hat{V}$ and **o**^*N*_*j*_^ are the output data, meaning **o**^*NJ*^ = *γ*. In this study, a rectified linear unit *ξ* (*x*) =  ^*J*^max (*x*, 0) was used as the activation function. It should be noted that *Ξ* is determined by the weights *𝕎*≔{{W_*m*,*n*_^1^}_*n*=1,⋯,*N*_1__^*m*=1,⋯,*N*_2_^, {*W*_*m*,*n*_^2^}_*n*=1,⋯,*N*_2__^*m*=1,⋯,*N*_3_^, ⋯, {*W*_*m*,*n*_^*J*−1^}_*n*=1,⋯,*N*_*J*−1__^*m*=1,⋯,*N*_*J*_^}. Therefore, we denote $\Xi \text{W}\left(\hat{V}\right)≔\Xi \left(\hat{V}\right)$. To determine W, we minimize the function

(14) $$\begin{array}{c} W≔\underset{W}{\arg\min}\overset{N_{T}}{\underset{k=1}{\sum }}{\left\|\Xi_{W}\left({\hat{V}}^{\left(k\right)}\right)-\gamma^{\left(k\right)}\right\|}^{2}, \end{array}$$

for a given training dataset $\left(\gamma^{\left(1\right)},{\hat{V}}^{\left(1\right)}\right)\left(\gamma^{\left(2\right)},{\hat{V}}^{\left(2\right)}\right),\cdots ,\left(\gamma^{\left(N_{T}\right)},{\hat{V}}^{\left(N_{T}\right)}\right)$, where *N*_*T*_ is the number of training data. As shown in (14), the set of weights *𝕎*, meaning the map *Ξ* is determined by the training dataset. In the next section, we will present the training dataset adopted in this study.

### 3.3. Training Dataset from Numerical Simulations for Constructing the Map *Ξ*

The map *Ξ* used in (12) and (13) is determined by *𝕎*, which is defined by the minimization in (14). The result of the minimization in (14) depends on the training dataset ${\left\{\left(\gamma^{\left(i\right)},{\hat{V}}^{\left(i\right)}\right)\right\}}_{i=1}^{N_{T}}$. Therefore, the map *Ξ* is ultimately determined by the training dataset. Consequently, the design of the training dataset is very important. Therefore, in this section, we present the details regarding how the training data were defined.

**Figure 5:**
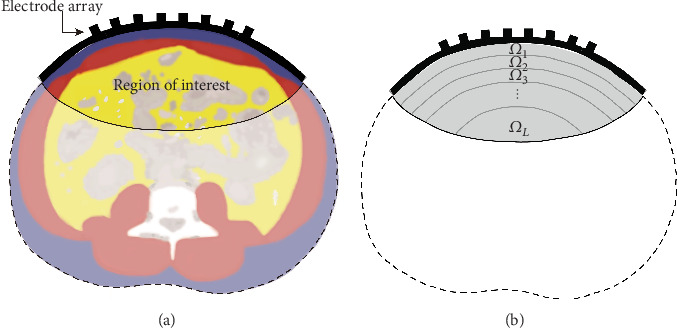
Region of interest in the imaging domain (a) and subregions of layers (b).

Generating training data requires solving equation (2) to derive the voltage data **V** that determine *g* and the conductivity distributionb *γ*. We adopted the body shape in (5) and the normalized voltage $\hat{V}$ in (11) for a given domain *Ω*, the electrode positions for the domain *Ω* from two-dimensional abdomen CT axial images, and considered a case with 10 electrodes on the central front part of the abdomen. According to [11], it is acceptable to consider a limited region around the electrode array, as shown in Figure 5, because measured voltages are rarely affected by the conductivity far from the electrodes. In the restricted region, we use a special type of internal domain that enables us to estimate the thickness of abdominal fat more easily. We propose dividing the imaging domain into *L* disjoint layers *Ω*_1_, ⋯, *Ω*_*L*_ with thicknesses of *d*_0_ such that

(15) $$\begin{array}{c} \Omega_{\ell }≔\left\{r\in \Omega :d_{0}\left(\ell -1\right)<\text{dist}\left(r,\partial \Omega \right)<d_{0}\ell \right\}\text{ and }\Omega_{L}≔\Omega \backslash \cup_{\ell =1}^{L-1}\Omega_{\ell }, \end{array}$$

for *ℓ* = 1, 2, ⋯, *L* − 1, where dist (**r**, *∂Ω*) denotes the distance between **r** and *∂Ω*. In this study, we used 15 thin layers (i.e., *L* = 15). By using the layers *Ω*, we can simply divide the domain *Ω* into three regions of subcutaneous fat, muscle, and other tissues, whose conductivity values are denoted as *γ*_*f*_, *γ*_*m*_, and *γ*_*r*_, respectively. Then, the conductivity distribution can be expressed as

(16) $$\begin{array}{c} \gamma \left(\text{r}\right)=\left\{\begin{array}{c} \gamma_{f},\text{r}\in \cup_{1}\leq \ell \leq \ell_{f}\Omega_{\ell },\\ \gamma_{m},\text{r}\in \cup_{\ell_{f}}\leq \ell \leq \ell_{m}\Omega_{\ell },\\ \gamma_{r},\text{otherwise}, \end{array}\right. \end{array}$$

where *ℓ*_*f*_ and *ℓ*_*m*_ are indices for the subregions of subcutaneous fat and muscle, respectively. It should be noted that *ℓ*_*f*_ < *ℓ*_*m*_ because the subcutaneous fat is the outermost region. Therefore, the thicknesses of the subcutaneous fat and muscle regions can be easily estimated as *d*_0_*ℓ*_*f*_ and *d*_0_(*ℓ*_*m*_ − *ℓ*_*f*_), respectively.

In this study, we tested all possible values of *ℓ*_*f*_ and *ℓ*_*m*_ while maintaining at least one layer for each region (i.e., minimum of *ℓ*_*f*_ = 1, maximum of *ℓ*_*f*_ = 13, minimum of *ℓ*_*m*_ = 2, and maximum of *ℓ*_*m*_ = 14). Therefore, we testing 91 different partitions for the three regions. Regarding the conductivity values for subcutaneous fat *γ*_*f*_, muscle *γ*_*m*_, and other tissues *γ*_*r*_, we assigned conductivity values ranging from 1 to 10 with a step size of 0.5 according to (1). Specifically, we tested *γ*_*f*_ = 1, *γ*_*m*_ = 2.0, 2.5, 3.0, ⋯, 10.0, and *γ*_*r*_ = 1.5, 2.0, 2.5, ⋯, 9.5 satisfying *γ*_*f*_ < *γ*_*r*_ < *γ*_*m*_ ≤ 10*γ*_*f*_. Therefore, we tested 17 different values for *γ*_*m*_ and nine different values for *γ*_*r*_. Considering the 91 different partitions for the three types of organs, a total of 13923( = 91 × 17 × 9) different conductivity distributions were used for the training dataset.

To produce a normalized voltage $\hat{V}$ with a given conductivity distribution *γ* in a given domain with a given electrode array, we used 45 different current application patterns (with all possible electrode pairs using 10 electrodes). For each application, we measured 28 voltages using all electrode pairs for which no current was applied. We used a set of pairs $\left(\gamma ,\hat{V}\right)$ of simplified conductivity distributions and normalized data for the training dataset.

By using the training dataset ${\left\{\left(\gamma^{\left(i\right)},{\hat{V}}^{\left(i\right)}\right)\right\}}_{i=1}^{N_{T}}$, we constructed the representation function *Ξ* defined in (12) using TensorFlow [43] with five hidden layers (*J* = 7). We set the numbers of nodes in each hidden layer as (*N*_2_, *N*_3_, *N*_4_, *N*_5_, *N*_6_) = (512, 256, 128, 64, 32).

## 4. Results

In this section, we present numerical simulations to validate the proposed method. In Section 4.1, we present reconstructed images of the human abdominal model to determine subcutaneous fat thickness, as well as conductivity values for different fat thicknesses and body shapes. To calculate the percentage error of thickness estimation, in Section 4.2, we present numerical simulations with various fat thicknesses in a circular domain. Additionally, numerical experiments in Section 4.3 demonstrate the robustness of the proposed reconstruction algorithm in a nonabdomen domain with random data and nonabdomen domain with regular data. Finally, in Section 4.4, we present a numerical validation of data normalization.

We present the image reconstruction algorithm below. To test the proposed algorithm, we normalized the measured data **V** to obtain $\hat{V}$ and inserted it into the function *Ξ*, resulting in the image $\gamma =\Xi \left(\hat{V}\right)$.

### 4.1. Image Reconstruction

**Figure 6:**
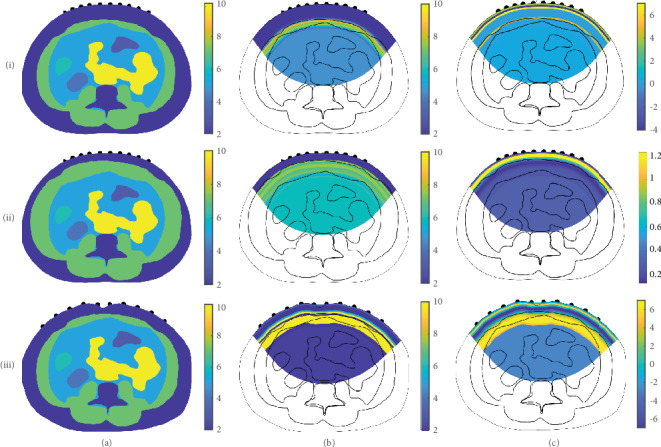
Numerical simulations for testing the proposed reconstruction algorithm with different subcutaneous fat and muscle thicknesses and body shapes. (i) Thick subcutaneous fat. (ii) Thin subcutaneous fat. (iii) Different body shape. (a) True conductivity distributions. (b) Reconstructed images generated by the proposed method. (c) Reconstructed images generated by the regularized least squares method.

**Figure 7:**
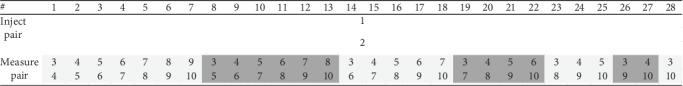
Electrode pairs for current application and voltage measurement when current is applied through the first and second electrodes.

This section presents the image reconstruction results of the proposed method in comparison to those of the conventional method (regularized least squares method), which was originally presented in (7). The first test image contains variations in the thicknesses of subcutaneous fat and muscle. For the second test image, we changed the body shape. To generate an internal conductivity distribution of the abdomen for our simulations, we used CT images and assigned conductivity values to each organ satisfying (1), as shown in Figure 6(a). To generate the current-voltage data **V** for (5), we applied 45( = 10 × 9 ÷ 2) currents to all possible pairs of electrodes. For each application, we measured 28 voltages between the remaining electrode pairs, where no current was applied (as shown in Figure 7).

**Figure 8:**
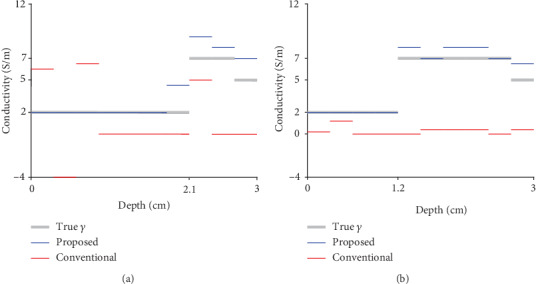
Profiles corresponding to the first and second rows of images in Figure 6. Each profile line begins at the top center of the corresponding image and moves to a vertical depth of up to 3 cm. In both plots, gray represents true conductivity values, and blue and red represent the reconstructed conductivity values generated by the proposed and conventional methods, respectively. (a) Thick fat case corresponding to the first row in Figure 6. (b) Thin fat case corresponding to the second row in Figure 6.

A total of 1260( = 45 × 28) voltages (*M*_∗_ = 1260) were used to reconstruct the conductivity distributions. The current-voltage data **V** was obtained by solving the governing equation (2) using the finite element method. In Figure 6, we present reconstructed images and ground-truth images. In Figure 8, we present profiles for ease of comparison.

In Figure 6, we present the results for varying subcutaneous fat and muscle thicknesses. The different types of test domains are arranged in row (i) for thick subcutaneous fat, row (ii) for thin subcutaneous fat, and row (iii) for a different body shape based on a different CT image. For comparison, we present images of (a) the true conductivity distribution and the reconstructed conductivity distributions generated by (b) the proposed method and (c) conventional method. We use the same color bar for the true conductivity distributions and the images reconstructed by the proposed method, and use an adjusted color bar to improve image contrast for the images reconstructed by the regularized least squares method. If we use the same color bar as the true images for the images reconstructed by the regularized least squares method, then the resulting images are almost homogeneous (i.e., no distinct image contrast). The images reconstructed by the proposed method contain distinct borders between subcutaneous fat and muscle, whereas the images reconstructed by the regularized least squares method fail to divide subcutaneous fat and muscle.

For ease of observing subcutaneous fat estimations, in Figure 8, we present profiles for the images in Figure 6. The profiles represent regions from the top center of each image to a vertical depth of up to 3 cm from each image. In Figure 8, we present both (a) thick fat and (b) thin fat cases corresponding to the first and second rows in Figure 6. In the case with thick fat, the proposed method succeeds in capturing the border between fat and muscle, whereas the conventional method cannot identify fat thickness and conductivity values. Furthermore, the proposed method can accurately reconstruct the conductivity value of fat. When the fat is thin, the proposed method is still able to estimate fat thickness and conductivity values, whereas the conventional method cannot determine fat thickness and conductivity values.

### 4.2. Thickness Estimation

**Figure 9:**
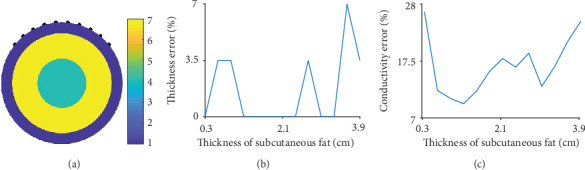
(a) One representative circular model for the test process among 13 models with subcutaneous fat thicknesses ranging from 0.3 to 3.9 cm. (b) Percentage errors of thickness estimation using the proposed method. (c) Percentage errors of conductivity estimation using the proposed method.

In this section, we present the percentage errors (relative errors of subcutaneous fat thickness as percentages) for estimating subcutaneous fat thickness and conductivity values using the proposed method (Section 3). To derive quantitative results, we used a circular model, rather than a body shape (for both training and testing), with a radius of 10 cm and various subcutaneous fat thicknesses ranging from 0.3 cm to 3.9 cm with a step size of 0.3 cm, as shown in Figure 9(a). The estimations of fat thickness were derived from the reconstructed concentric circles (layers) by measuring the length from the boundary to the border of each layer, where the conductivity values change abruptly. The results of estimating subcutaneous fat thickness are values corresponding to 13 thickness classes (possible fat thickness classes for the training model): 0.3, 0.6, 0.9, 1.2, 1.5, 1.8, 2.1, 2.4, 2.7, 3.0, 3.3, 3.6, and 3.90 cm. Because the testing model used the same variations in subcutaneous fat thickness as the training model, the errors are discretely distributed and can be equal to zero (Figure 9(b)). The conductivity values of fat were also estimated and the corresponding percentage errors are presented in Figure 9(c). The errors of estimating thickness and conductivity values are always less than 7% and 28%, respectively, for subcutaneous fat thicknesses ranging from 0.3 to 3.9 cm.

### 4.3. Robustness

The goal of this section is to demonstrate the robustness of the proposed method for nonabdominal data. Based on the results presented in this section, we can conclude that the proposed reconstruction algorithm does not artificially generate human abdomen images from nonabdominal data. For verification, we tested two types of data: (1) impedance data from abdominal shape domains with nonabdominal conductivity distributions and (2) data consisting of random numbers.

**Figure 10:**
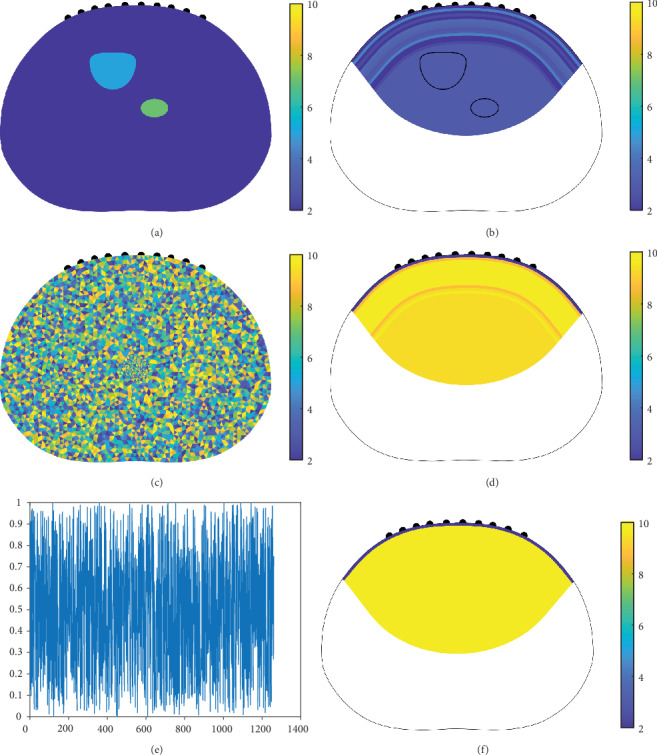
(a, c) True conductivity distributions. (b, d) Corresponding image reconstruction results generated by the proposed method. (e) Voltage data generated based on random numbers drawn from a Gaussian distribution. (f) Image reconstruction results generated by the proposed method.

We use the same abdominal domain shape and electrode alignment as those used in Section 4.1 and shown in Figure 6(i). The first impedance data we tested came from an abdominal shape domain with various conductivity anomalies. The background conductivity value was two, the upper anomalous conductivity value was seven, and the lower anomalous conductivity value was five, as shown in Figure 10(a). Next, we tested impedance data from an abdominal shape domain with random conductivity distributions drawn from a Gaussian distribution, as shown in Figure 10(c). The reconstruction results in Figures 10(b), 10(d), and 10(f) do not exhibit a fat-muscle structure and show almost entirely background conductivity values.

### 4.4. Data Normalization

**Figure 11:**
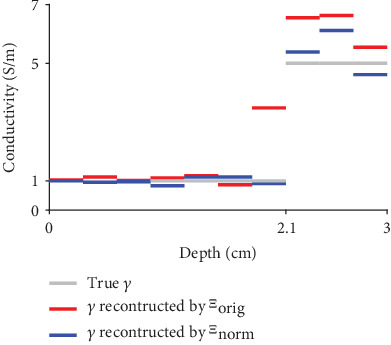
True and reconstructed conductivity values near the boundary and up to a depth of 3 cm. The gray line represents true conductivity values. The red and blue lines represent reconstructed conductivity values generated from *Ξ*_orig_ and *Ξ*_norm_ using **V** and $\hat{V}$, respectively. The results of using *Ξ*_orig_ fail to capture the border between the subcutaneous fat and muscle.

In this section, we present evidence of the benefits of using the normalized data **V** in (11), rather than using the data **V** in (5) with the same current measurement patterns discussed in Section 4.1. To this end, we compare the results of MLPs (Section 3.2) different geometrical shapes for the training process (circular model) and testing process trained using $\hat{V}$ and **V**. For the purpose of comparing geometrical influences, we used (elliptical model). In the training process, we used a circular model to create MLP maps *Ξ*_norm_ and *Ξ*_orig_ from $\hat{V}$ and **V**, respectively, to *γ* using the network in Figure 4. For the circular model, we maintained a radius of 10 cm and generated concentric circles for subcutaneous fat, muscle, and interior tissues. The first outer layer of the model was subcutaneous fat with thicknesses ranging from 0.3 cm to 3.9 cm. The second layer of the model was muscle with various thicknesses that satisfied the condition of the total thickness of fat and muscle being equal to 4.2 cm. We set the conductivity value of the subcutaneous fat to 1 S/m and tested various conductivity values for the muscle and interior tissues ranging from 2 to 10 S/m and from 1.5 to 9.5 S/m, respectively, satisfying the condition that the conductivity value of the muscle was always greater than that of the interior tissues. According to the settings described above, the total number of training data was 13923. For testing, we used an elliptical model with a major axis of 15 cm and a minor axis of 9 cm and a subcutaneous fat thickness of 2.1 cm. The other settings were the same as those used for the training data. We applied each MLP map (*Ξ*_norm_ and *Ξ*_orig_) to the elliptical model. For ease of comparison, Figure 11 presents conductivity profiles derived from the reconstructed images based on *Ξ*_norm_ and *Ξ*_orig_. The results of using *Ξ*_norm_ reveal accurate subcutaneous fat thicknesses, while those of using *Ξ*_orig_ are inaccurate, as shown in Figure 11.

### 4.5. Phantom Experiments

**Figure 12:**
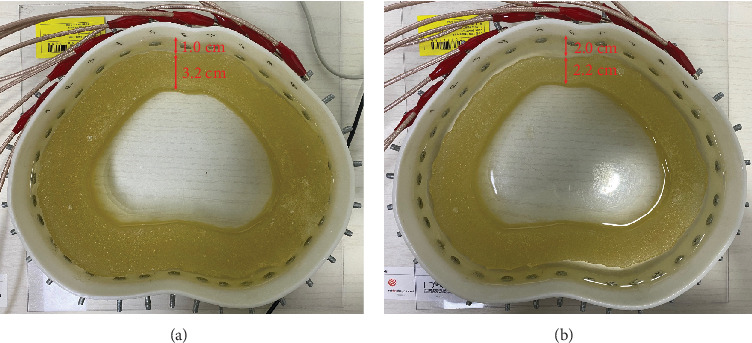
Abdomen-shaped phantom with agars of different thicknesses. (a) 3.2 cm of the thickness of the agar and 1 cm of the distance to the boundary. (b) 2.2 cm of the thickness of the agar and 2 cm of the distance to the boundary.

**Figure 13:**
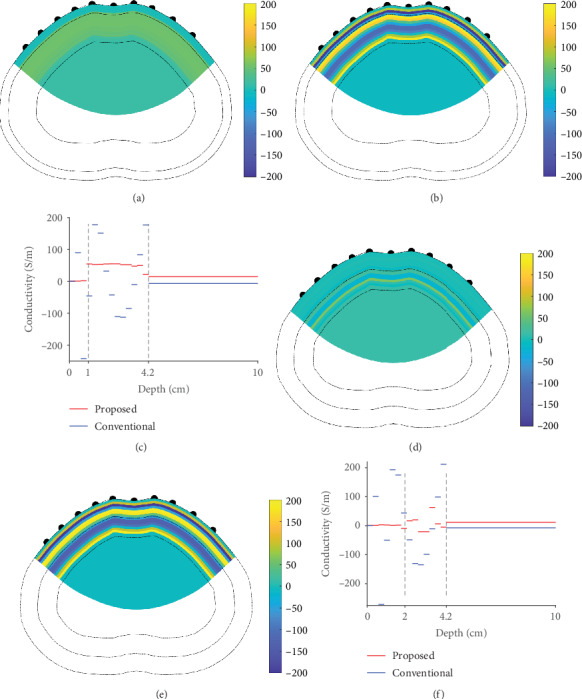
Reconstructed images from phantom experiments using the proposed and conventional methods. Reconstructed images for the 1 cm fat layer generated by the (a) proposed method and (b) conventional method. (c) Profile of the reconstructed conductivity values along the depth direction. For the 3.2 cm fat layer, reconstructed images generated by the (d) proposed method and (e) conventional method.

In this section, we present phantom experiments to validate the proposed method. We used a specially designed phantom with a boundary shape representing the human abdomen and attached 10 electrodes to the front of the phantom, as shown in Figure 12(a). To generate conductivity distributions inside the phantom, we placed a toroidal agar fabricated from gelatin away from the boundary of the phantom at a uniform distance from the boundary in all directions. We used a saline solution (0.1% NaCl) to fill the two regions separated by the agar, namely, the near boundary and the middle of the phantom. Here, the agar represents the muscle layer and the regions separated by the agar represent the regions of subcutaneous fat and the interior organs. The electrical conductivity values of the agar and saline water are 9.2 S/m and 0.2 S/m, respectively. The thickness of the agar is 3.2 cm and the distance from the boundary is 1 cm, meaning the thicknesses of the muscle and fat layers are 3.2 cm and 1 cm, respectively. We used a Sciospec 16 channel EIT system to apply 45 currents and measured 28 voltages for each current application, as described in Section 4.1. The amount of current applied was 8 mA (as peak amplitude) with an excitation frequency of 100 kHz. We applied the proposed method and conventional method to the measured data from the phantom for image reconstruction. To consider a more obese abdomen case, we reduced the thickness of the agar to 1 cm by trimming the outer section of the agar, resulting in a distance from the agar to the boundary of 3.2 cm, as shown in Figure 12(b). In Figure 13, we present the image reconstruction results for the phantom experiments. In the case with a thin fat layer (1 cm), the conductivity distribution of the proposed method abruptly changes at the borders, whereas the conductivity distribution of the conventional method does not reveal a clear border between fat and muscle. In the case with a thick fat layer (2 cm), the reconstructed conductivity values in the fat region are constant for the proposed method, but the conventional method yields irregular reconstructed conductivity values in the fat region. These results demonstrate that both the thicknesses and conductivity distributions of fat layers can be more clearly identified by the proposed method compared to the conventional method. However, the reconstructed conductivity values at the fat region are overestimated.

## 5. Conclusions and Discussion

We proposed an absolute EIT reconstruction method for abdominal fat estimation using an MLP, which is a deep-learning technique. The absolute EIT problem is nonlinear, ill-posed, and severely affected by forward modelling errors stemming from uncertainty in electrode positions and body geometry. We adapted an MLP to capture the nonlinear relationships between current-voltage data and conductivity distributions. To alleviate forward modelling errors, we normalized the current-voltage data based on information regarding electrode positions and body geometry. When performing reconstruction, we separated the problem domain into sublayers of uniform thickness to facilitate the estimation of abdominal fat thickness. This specially designed separation of the problem domain significantly reduced the number of unknown variables, thereby reducing the ill-posedness of the absolute EIT problem. We validated the proposed method through numerical simulations and phantom experiments using 10 channel EIT systems with a human-like domain and varying thicknesses of fat and muscle.

Based on the presence of errors in the data **V**, a wider domain should be considered for stable reconstruction of the conductivity distribution *γ* as follows:

(17) $$\begin{array}{c} {ϒ}_{\varepsilon }≔\left\{\gamma :\left\|S_{\gamma }\gamma -V\right\|<\varepsilon \right\}, \end{array}$$

where *ε* > 0 is the error tolerance. Based on the ill-posed nature of EIT, small errors in the data can result in abrupt changes in outputs (diam(*ϒ*_*ε*_) ≫ 1). The regularizations in (7) can be considered as an attempt to restrict the wide domain **Y**_*ε*_ by using prior knowledge regarding sparsity (*L*_1_), smoothness (*L*_2_), and sharpness (total variation), but these regularizations cannot completely eliminate infeasible solutions, meaning they cannot guarantee stable absolute EIT reconstruction. In the proposed deep learning method, the restriction of **Y**_*ε*_ can be achieved easily to reject infeasible solutions by designing suitable training data. Specifically, one should only select feasible conductivity distributions for the training set to satisfy the desired properties for restriction.

The fully connected nature of MLP layers may be redundant because boundary voltage is insensitive to local perturbations in conductivity, meaning the entanglement between boundary voltages and conductivity is weak at regions far from electrodes.

One could use partially connected layers. This method is typically referred to as a convolutional neural network [44]. The use of partially connected layers reduces the computational cost of the training process because it requires fewer weights to be optimized.

In this study, we assumed that the thickness of subcutaneous fat was uniform when constructing training data. This model not only makes estimation of the thickness of subcutaneous fat easier, but also makes the problem less ill-posed by reducing the number of unknown variables. However, the proposed method may be less accurate when the thickness of subcutaneous fat is nonuniform.

## Figures and Tables

**Algorithm 1:**
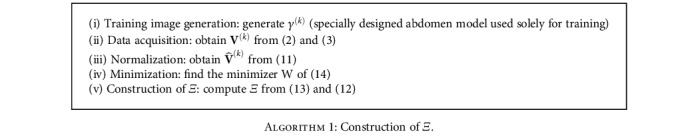
Construction of *Ξ*.

## Data Availability

The Matlab data used to support the findings of this study are available from the corresponding author upon request.

## References

[1] Després J., Lemieux I. (2006). Abdominal obesity and metabolic syndrome. *Nature*.

[2] Després J., Lemieux I., Bergeron J. (2008). Abdominal obesity and the metabolic syndrome: contribution to global cardiometabolic risk. *Arteriosclerosis, Thrombosis, and Vascular Biology*.

[3] Ritchie S. A., Connell J. M. C. (2007). The link between abdominal obesity, metabolic syndrome and cardiovascular disease. *Metabolism & Cardiovascular Diseases*.

[4] Shen W., Punyanitya M., Wang Z. (2004). Total body skeletal muscle and adipose tissue volumes: estimation from a single abdominal cross-sectional image. *Journal of Applied Physiology*.

[5] Sodickson A., Baeyens P. F., Andriole K. P. (2009). Recurrent CT, cumulative radiation exposure, and associated radiation-induced cancer risks from CT of adults. *Radiology*.

[6] Holder D. S. (2005). *Electrical impedance tomography: methods, history and applications*.

[7] Seo J. K., Woo E. J. (2013). *Nonlinear Inverse Problems in Imaging*.

[8] Adler A., Grychtol B., Bayford R. (2015). Why is EIT so hard, and what are we doing about it?. *Physiological Measurement*.

[9] Yamaguchi T., Maki K., Katashima M. (2010). Practical human abdominal fat imaging utilizing electrical impedance tomography. *Physiological Measurement*.

[10] Yamaguchi T. F., Katashima M., Wang L., Kuriki S. (2012). Improvement of image reconstruction of human abdominal conductivity by impedance tomography considering the bioelectrical anisotropy. *Advanced Biomedical Engineering*.

[11] Ammari H., Kwon H., Lee S., Seo J. K. (2017). Mathematical framework for abdominal electrical impedance tomography to assess fatness. *SIAM Journal on Imaging Sciences*.

[12] Lionheart W. R. B., Kaipio J., McLeod C. N. (2001). Generalized optimal current patterns and electrical safety in EIT. *Physiological Measurement*.

[13] Calderón A. P. (1980). On an inverse boundary value problem in seminar on numerical analysis and its applications to continuum physics. *Brazilian Mathematical Society*.

[14] Kohn R., Vogelius M. (1984). Determining conductivity by boundary measurements. *Communications on Pure and Applied Mathematics*.

[15] Sylvester J., Uhlmann G. M. (1986). A uniqueness theorem for an inverse boundary value problem in electrical prospection. *Communications on Pure and Applied Mathematics*.

[16] Cheney M. (1999). Electrical impedance tomography. *SIAM Review*.

[17] Liu D., Kolehmainen V., Siltanen S., Laukkanen A., Seppänen A. (2015). Estimation of conductivity changes in a region of interest with electrical impedance tomography. *Inverse Problems and Imaging*.

[18] Santosa F., Michael V. (1990). A backprojection algorithm for electrical impedance imaging. *SIAM Journal on Applied Mathematics*.

[19] Cheney M., Isaacson D., Newell J., Simske S., Goble J. (1990). NOSER: an algorithm for solving the inverse conductivity problem. *International Journal of Imaging Systems and Technology*.

[20] Adler A., Arnold J. H., Bayford R. (2009). GREIT: a unified approach to 2D linear EIT reconstruction of lung images. *Physiological measurement*.

[21] Siltanen S., Jennifer M., David I. (2000). An implementation of the reconstruction algorithm of A Nachman for the 2D inverse conductivity problem. *Inverse Problems*.

[22] Brühl M., Martin H. (2000). Numerical implementation of two noniterative methods for locating inclusions by impedance tomography. *Inverse Problems*.

[23] Hua P., Woo E. J., Webster J. G., Tompkins W. J. (1991). Iterative reconstruction methods using regularization and optimal current patterns in electrical impedance tomography. *IEEE Transactions on Medical Imaging*.

[24] Hamilton S., Lionheart W., Adler A. (2019). Comparing D-bar and common regularization-based methods for electrical impedance tomography. *Physiological Measurement*.

[25] Knudsen K., Lassas M., Mueller J. L., Siltanen S. (2007). D‐Bar method for electrical impedance tomography with discontinuous conductivities. *SIAM Journal on Applied Mathematics*.

[26] Knudsen K., Lassas M., Mueller J. (2009). Regularized D-bar method for the inverse conductivity problem. *Inverse Problems & Imaging*.

[27] Hamilton S. J., Hänninen A., Hauptmann A., Kolehmainen V. (2019). Beltrami-net: domain-independent deep D-bar learning for absolute imaging with electrical impedance tomography (a-EIT). *Physiological Measurement*.

[28] Hauptmann M., Santacesaria M., Siltanen S. (2017). Direct inversion from partial-boundary data in electrical impedance tomography. *Inverse Problems*.

[29] Biguri A., Adler A., Grychtol B., Soleimani M. (2015). Tracking boundary movement and exterior shape modelling in lung EIT imaging. *Physiological Measurement*.

[30] Nissinen A., Kolehmainen V. P., Kaipio J. P. (2011). Compensation of modelling errors due to unknown domain boundary in electrical impedance tomography. *IEEE Transactions on Medical Imaging*.

[31] Dardé J., Hyvönen N., Seppänen A., Staboulis S. (2013). Simultaneous recovery of admittivity and body shape in electrical impedance tomography: an experimental evaluation. *Inverse Problems*.

[32] Hamilton S. J., Mueller J. L., Santos T. R. (2018). Robust computation in 2D absolute EIT (a-EIT) using D-bar methods with the ‘exp’ approximation. *Physiological Measurement*.

[33] Ali T., Ling S. H. (2019). Review on electrical impedance tomography: artificial intelligence methods and its applications. *Algorithms*.

[34] Hamilton S., Andreas H. (2018). Deep D-Bar: real-time electrical impedance tomography imaging with deep neural networks. *IEEE Transactions on Medical Imaging*.

[35] Martin S., Choi C. T. M. (2015). Nonlinear electrical impedance tomography reconstruction using artificial neural networks and particle swarm optimization. *IEEE Transactions on Magnetics*.

[36] Seo J. K., Kim K. C., Jargal A., Lee K., Harrach B. (2019). A learning-based method for solving ill-posed nonlinear inverse problems: a simulation study of lung EIT. *EIT SIAM Journal on Imaging Sciences*.

[37] Wallner G. (2019). *Data Analytics Applications in Gaming and Entertainment*.

[38] Hasgall P. A., Di Gennaro F., Baumgartner C. IT’IS Database for thermal and electromagnetic parameters of biological tissues. https://itis.swiss/database/.

[39] Kolehmainen V., Vauhkonen M., Karjalainen P. A., Kaipio J. P. (1997). Assessment of errors in static electrical impedance tomography with adjacent and trigonometric current patterns. *Physiological Measurement*.

[40] Lionheart W. R. B. (2004). EIT reconstruction algorithms: pitfalls, challenges and recent developments. *Physiological Measurement*.

[41] LeCun Y. (2015). Deep learning. *Nature*.

[42] Schmidhuber J. (2015). Deep learning in neural networks: an overview. *Neural Networks*.

[43] Abadi M., Agarwal A., Barham P. (2015). TensorFlow: large-scale deep learning on heterogeneous systems. https://www.tensorflow.org/.

[44] Sze V., Chen Y.-H., Yang T.-J., Emer J. S. (2017). Efficient processing of deep neural networks: A tutorial and survey. *Proceedings of the IEEE*.

